# GDF-15 Suppresses Puromycin Aminonucleoside-Induced Podocyte Injury by Reducing Endoplasmic Reticulum Stress and Glomerular Inflammation

**DOI:** 10.3390/cells13070637

**Published:** 2024-04-05

**Authors:** Ekaterina von Rauchhaupt, Martin Klaus, Andrea Ribeiro, Mohsen Honarpisheh, Chenyu Li, Min Liu, Paulina Köhler, Karina Adamowicz, Christoph Schmaderer, Maja Lindenmeyer, Stefanie Steiger, Hans-Joachim Anders, Maciej Lech

**Affiliations:** 1 Department of Medicine IV, Renal Division, Ludwig-Maximilians-University Hospital, Ludwig-Maximilians-University Munich, 80336 Munich, Germany; katjats95@gmail.com (E.v.R.); martin.klaus@med.uni-muenchen.de (M.K.); andrea.lima@med.uni-muenchen.de (A.R.); mohsen.honarpisheh@med.uni-muenchen.de (M.H.); chenyu.li@med.uni-muenchen.de (C.L.); min.liu@med.uni-muenchen.de (M.L.); koehlerpaulina@gmail.com (P.K.); stefanie.steiger@med.uni-muenchen.de (S.S.); hjanders@med.uni-muenchen.de (H.-J.A.); 2 Klinikum Rechts der Isar, Department of Nephrology, Technical University Munich, 81675 Munich, Germany; christoph.schmaderer@mri.tum.de; 3 Department of Microbiology, Faculty of Biochemistry, Biophysics and Biotechnology of Jagiellonian University, 30-387 Krakow, Poland; karina.adamowicz@uj.edu.pl; 4 III Department of Medicine, University Medical Center Hamburg-Eppendorf, 20251 Hamburg, Germany; m.lindenmeyer@uke.de

**Keywords:** GDF15, podocytes, endoplasmic reticulum stress, podocytopathies, glomerular inflammation

## Abstract

GDF15, also known as MIC1, is a member of the TGF-beta superfamily. Previous studies reported elevated serum levels of GDF15 in patients with kidney disorder, and its association with kidney disease progression, while other studies identified GDF15 to have protective effects. To investigate the potential protective role of GDF15 on podocytes, we first performed in vitro studies using a *Gdf15*-deficient podocyte cell line. The lack of GDF15 intensified puromycin aminonucleoside (PAN)-triggered endoplasmic reticulum stress and induced cell death in cultivated podocytes. This was evidenced by elevated expressions of *Xbp1* and ER-associated chaperones, alongside AnnexinV/PI staining and LDH release. Additionally, we subjected mice to nephrotoxic PAN treatment. Our observations revealed a noteworthy increase in both GDF15 expression and secretion subsequent to PAN administration. *Gdf15* knockout mice displayed a moderate loss of WT1+ cells (podocytes) in the glomeruli compared to wild-type controls. However, this finding could not be substantiated through digital evaluation. The parameters of kidney function, including serum BUN, creatinine, and albumin–creatinine ratio (ACR), were increased in *Gdf15* knockout mice as compared to wild-type mice upon PAN treatment. This was associated with an increase in the number of glomerular macrophages, neutrophils, inflammatory cytokines, and chemokines in *Gdf15*-deficient mice. In summary, our findings unveil a novel renoprotective effect of GDF15 during kidney injury and inflammation by promoting podocyte survival and regulating endoplasmic reticulum stress in podocytes, and, subsequently, the infiltration of inflammatory cells via paracrine effects on surrounding glomerular cells.

## 1. Introduction

Podocytopathies are podocyte-injury-related kidney diseases that can manifest with mild proteinuria or nephrotic syndrome. The development and severity of these diseases are influenced by a combination of genetic predispositions and environmental triggers, including factors related to inflammation and infection. Kidney biopsies of patients display various lesions ranging from a minimal change (MC) pattern to diffuse mesangial sclerosis (DMS) and focal segmental glomerulosclerosis (FSGS). These lesions represent different patterns of podocyte injury without indicating a specific trigger of the damage. For instance, minimal change disease (MCD) refers to the absence of significant histological changes observed under light microscopy, despite the loss of the podocyte ultrastructure [1]. However, steroid non-responders are in danger of disease progression to FSGS driven by ongoing podocyte injury [1]. The prevalence and combination of inflammatory, infectious environmental agents contributing to podocyte injury and loss vary throughout an individual’s lifespan. Numerous genes associated with hereditary podocytopathies have been identified, with more than 50 genes currently known. Mutations in genes such as *NPHS1* (encoding nephrin), *NPHS2* (encoding podocin), *ACTN4* (encoding α-actin 4), and *ANLN* (encoding anillin) have highlighted the importance of the slit diaphragm and podocyte actin cytoskeleton in kidney physiology [2]. Moreover, various studies focusing on podocytopathies have emphasized common acquired factors such as inflammation, mitochondrial injury, and metabolic disorders as contributing factors [3]. In addition, endoplasmic reticulum (ER) stress has been widely demonstrated to contribute to podocyte injury [4,5,6]. For instance, APOL1 risk variants contribute to podocyte injury through enhanced ER stress [7].

Growth-Differentiation-Factor 15 (GDF15), also known as macrophage inhibitory cytokine-1 (MIC1), was initially been identified as a distinct member of the TGFβ superfamily with a high expression in placenta tissue, leading to its alternative name as placenta bone morphogenetic protein (PLAB) [8]. Despite structural similarities among TGFβ family members, GDF15 exhibits significant amino acid sequence differences and unique biological functions [9]. For instance, it shares around 30% sequence homology with TGF-β1, suggesting a distinct biologic activity [10,11]. GDF15 concentrations rise in various diseases, including metastatic prostate, breast, and colorectal carcinomas, anorexia/cachexia, and cardiovascular injury [12,13,14,15]. Different immune cell types, such as macrophages, show the increased expression of GDF15 in response to tissue injury, pro-inflammatory mediators, and pathogen/damage-associated molecular patterns [16,17]. Studies in murine models have shown a role for GDF15 in inflammatory diseases. For example, in sepsis, GDF15 improves survival by promoting tissue tolerance against inflammatory damage [18]; in heart ischemia and myocardial infarction, GDF15 exhibits anti-apoptotic [15] and inflammatory effects by interfering with chemokine signaling and integrin activation in neutrophils [15]. In the kidney during anti-GBM nephritis, GDF15 regulates T-cell numbers via a CXCR3-dependent mechanism [19]. Furthermore, our recent data revealed that GDF15 functions as a suppressor of lymphoproliferation, B- and T-cell expansion, and autoreactive plasma-cell maturation in an autoimmune model, which resulted in reduced anti-DNA-directed autoantibody production, possibly through TLR-7/-9 hyperresponsiveness in the exaggerated type I interferon (IFN-I) signature observed in *Gdf15*-deficient lupus-prone mice [17]. In patients with focal segmental glomerulosclerosis (FSGS), GDF15 is significantly upregulated, suggesting a crucial role for GDF15 in the pathogenesis of podocytopathies. Arif et al. proposed GDF15 as a primary gene involved in the pathogenesis of podocytopathy, as it is elevated in response to podocyte injury [20].

Considering the anti-inflammatory effects of GDF15 in other cell types and podocyte loss as characteristic feature podocytopathies, we hypothesized that GDF15 may play a renoprotective role during podocyte injury induced by puromycin aminonucleoside (PAN). This model offers distinct advantages in studying podocyte injury and associated mechanisms. PAN-induced injury primarily targets podocytes directly, providing insights into podocyte-specific responses to injury and potential therapeutic interventions. Our findings identified GDF15 as a novel glomeruli-acting protective factor both in vitro and in vivo.

## 2. Materials and Methods

Animal experiments: *Gdf15* knockout strain (MGI: 2386300, Gdf15tm1Sjl) [21] with the C57BL/6 background and wild-type C57BL/6 mice were used in experiments. Gdf15 knockout mice born with Mendelian ratios showed normal litter sizes. Each group of five mice was accommodated in individually ventilated cages, ensuring sterile conditions. They were kept under a 12 h light/dark cycle and offered autoclaved food and water ad libitum. Our experiments involved male and female mice aged 6–7 weeks. This study was conducted in compliance with the principles outlined in Directive 2010/63/EU on the Protection of Animals Used for Scientific Purposes (55.2-1-54-2532-63-12; LKE 258/2020).

Induction of renal injury and collection of tissues: Mice aged 6–7 weeks received an intraperitoneal injection of 400 mg/kg PAN (Sigma-Aldrich, Taufkirchen, Germany) to induce kidney injury, while a control group received injections of phosphate-buffered saline (PBS). Urine samples were collected 24 h and 7 days after PAN injection. On the seventh day post-injection, mice were euthanized via cervical dislocation, and blood and kidneys were collected. Tissues from each mouse were utilized for histological analysis, with additional samples stored at −80 °C for gene expression analysis.

Histological analysis and kidney function parameter: The kidneys were preserved in 4% buffered formalin, undergoing overnight processing (dehydration and clearing) in a tissue processor, and embedded in paraffin. We employed specified antibodies as per the manufacturer’s recommendations: Mac2 (Cat. No. 125402 from BioLegend, Koblenz, Germany, 1:3000); WT-1 (MA5-32215, Invitrogen, Darmstadt, Germany, 1:100); leukocytes Ly-6B.2 (1:100, Serotec, Kidlington, UK); and T lymphocytes were visualized by rat anti-mouse CD3 antibody (1:400 Serotec, Kidlington, UK). Manual analysis involved examining at least 20 glomeruli per kidney. Additionally, a deep-learning-assisted morphometry analysis of whole-slide images was conducted. To this end, histopathological slides were digitalized using an Aperio GT 450 DX scanner (Leica, Wetzlar, Germany). Slide annotations were created in QuPath v0.4.4 [22]. A customized deep-learning segmentation pipeline using Tensorflow (v2.14.0) was implemented and trained, and obtained segmentations of glomeruli and cells were manually corrected if necessary.

We assessed serum and urinary creatinine levels using the Jaffe method and blood urea nitrogen (BUN) utilizing an enzymatic test, following the manufacturer’s instructions (Diasys, Holzheim, Germany). Albuminuria was determined using mouse Albumin Quantification Set (Bethyl Laboratories, Montgomery, TX, USA) and proteinuria was assessed via Bradford method. We calculated urinary albumin–creatinine ratio (ACR) from urine samples as an indicator of significant albuminuria. This approach was chosen to avoid the need for 24 h urine collection in a metabolic cage [23].

RNA extraction, reverse transcription, and qRT-PCR: Quantitative real-time PCR (qRT-PCR) from cDNA was conducted using a Light Cycler 480 (Roche, Penzberg, Germany) with SYBR Green Dye detection. Gene-specific primers (225 nM, Metabion, Martinsried, Germany) were utilized. Standard controls were incorporated to ensure reliable results, assessing genomic DNA contamination, RNA quality, and overall PCR performance. The 2ΔCT method quantified target gene expression in each sample by comparing CT values of reference and target genes. We assessed the ΔCT value for each sample by subtracting the CT of the target gene from the CT of the reference gene. We calculated the 2ΔCT value by raising 2 to the power of the ΔCT value. We obtained the relative expression level of the target genes (not fold induction) allowing for better comparison of expression levels among different genes within the same sample. RNA isolation employed the Norgen Biotek Total RNA Purification kit (Thorold, ON, Canada) and MagNA Lyser Green beads (Roche, Basel, Switzerland). Total RNA was quantified with a NanoDrop ND-1000 Spectrophotometer. For cDNA conversion, 1 µg of total RNA underwent reverse transcription. Controls with ddH2O were negative. Melting curve profiles and agarose gel visualization ensured specificity. All primers were from Metabion (Martinsried, Germany).

In vitro cell experiments: Mouse podocyte cell line was provided by P. Mundel (Department of Medicine, University of Miami, Miller School of Medicine, Miami, FL, USA) and was cultured as previously described [24]. Shortly, the cells were cultured on collagen A-coated plates at 33 °C in RPMI supplemented with 10% FCS, 1% PS, and 100 ng/mL mouse recombinant IFN-g (Immunotools, Friesoythe, Germany). For the purpose of differentiation and subsequent experiments, 100,000 cells were seeded in 0.5 mL medium in 12-well plates or 300,000 cells were seeded in 1 mL medium in 6-well plates. In the case of differentiation experiments, the medium used did not contain IFN-g. The cells were used on day 10. To assess cytotoxicity and cell death, we employed the in vitro lactate dehydrogenase (LDH) assay (Roche, Penzberg, Germany), following the manufacturer’s guidelines. In brief, 10,000 cells were seeded per well in a 96-well plate using complete media and incubated overnight. Cells were stimulated with PAN 50µg/mL for 24 h, 48 h, or 72 h. Cell viability and metabolic activity were measured with MTT (3-(4,5-dimethylthiazol-2-yl)-2,5-diphenyltetrazolium bromide) method following the manufacturer’s instruction. Shortly, 40,000 cells were seeded per well in a 96-well plate in complete media overnight. Then, the medium was changed with 0% FCS as negative control and 2%, 10%, and 20% as positive controls for 72 h. Samples were incubated with MTT for 2 h, stopped, and measured at 570 nm. Primary tubular epithelial cells were isolated from the kidneys of BL6 mice. Isolated primary cells were cultured in DMEM/F12 with 10% FCS, 1% penicillin-streptomycin, 125 ng/mL PG E1 (Calbiochem, Darmstadt, Germany), 25 ng/mL EGF, 1.8 μg/mL l-thyroxine, 3.38 ng/mL hydrocortisone, and 2.5 mg/mL of insulin-transferrin-sodium selenite supplement (Sigma-Aldrich, Taufkirchen, Germany). MTCs, also known as MRPTEpiC, were cultured in DMEM/F12 (GIBCO, Invitrogen, CA, USA) with 10% fetal calf serum, and 1% penicillin–streptomycin. BMDM and BMDC: Bone marrow was collected from the femur and tibia of BL6 mice by flushing the bones with PBS. Collected cells were washed with PBS. After the washing step, cells were cultured in DMEM 10% FBS with 10 ng of MCSF (bone-marrow-derived macrophage) or 50 ng of GM-CSF (bone-marrow-derived dendritic cells) for 7 days.

Plasmids and CRISPR/Cas9-based gene KO: To knock out GDF15 gene in murine podocytes, guide RNAs were designed using the Design Tool Benchling database [25]. Four gRNAs binding to exon 1 were selected based on off-target and on-target activity. Single-strand gRNA (Metabion, Martinsried, Germany) were cloned as described previously [26]. Briefly, top and bottom of gRNA were incubated with T4 PNK enzyme (New England BioLabs, Frankfurt am Main, Germany) to generate double-strand gRNA. Double-strand oligo DNA for each gRNA was cloned into the BbsI site of the SpCas9-2A-GFP plasmid (PX458, Addgene, Cambridge, MA, USA) for expressing sgRNA and Cas9. Digestion and ligation reaction was set up with FastDigest BbsI (Fermentas, Rockville, MD, USA), T4 ligase (New England BioLabs, Frankfurt am Main, Germany), DTT, ATP, and Tango buffer (Fermentas, Rockville, MD, USA). The reaction was incubated for 5 min at 37 °C and 21 °C with 6 cycles for 1 h. The reaction was subjected to PlasmidSafe exonuclease to digest any residual linearized DNA. After transformation and purification, the positive clones were sent for sequencing to confirm gRNA insert. Podocytes were transfected with the expression plasmids using Lipofectamin 2000. Individual GFP-positive cells were sorted as single cells using flow cytometry and grown into a new clonal cell line, and tested by ELISA for production of GDF15. Non-KO clones transfected with empty plasmid were used as control cells.

Protein assays: The Mouse GDF15 ELISA (DuoSet ELISA, R&D Systems, DY6385, Minneapolis, MN, USA) was performed using supernatants obtained from 2 × 10^4^ cultured cells according to manufacturer instructions. For cytokine analysis of serum from mice or cell culture experiments, samples were prepared following the instructions of the BD Cytometric Bead Array Mouse Inflammation Kit. The quantification of cytokines in the samples was performed using the FCAP software V2. For Western blotting, protein samples were prepared by lysing cells or tissues in a lysis buffer containing protease inhibitors, denatured by heating at 95 °C, separated by gel electrophoresis, and transferred onto a PVDF membrane using electroblotting. The membrane was blocked with 5% non-fat milk, followed by incubation with primary antibodies at 4 °C overnight, subsequent washes, and incubation with secondary HRP-antibodies for 1 h at room temperature. To document the loading controls, the membrane was reprobed with a primary antibody against the housekeeping protein β-actin. The primary antibodies used were as follows: anti-PARP1 (Cell Signaling Technology, Danvers, MA, USA, Cat# 9542, 1:1000 for Western blot); anti-β-actin (Cell Signaling Technology, Cat# 4970, 1:10,000 for Western blot).

Statistical analysis: The data prepared in GraphPad Prism software (latest v. 10.2.2) was expressed as mean ± SD. The Mann–Whitney U test was employed for direct comparisons between two groups i.e., wild-type and knockout cells/mice, due to small sample size and non-parametric distribution of data (assessed via D’Agostino–Pearson normality test). Student’s *t* test was used for direct comparisons between single groups, i.e., wild-type and knockout cells/mice, in case of normally distributed data or sample size n > 15. The one-way analysis of variance (ANOVA) was conducted for statistics involving three or more independent (unrelated) groups. Tukey’s test was applied for all-possible pairwise comparisons. Statistical significance was indicated as follows: *p* value of <0.05 (*); *p* value of <0.01 (**); and *p* value of <0.001 (***).

## 3. Results

### 3.1. Podocytes Express and Secrete GDF15 and Its Deficiency Correlates with Podocyte Loss

**Figure 1:**
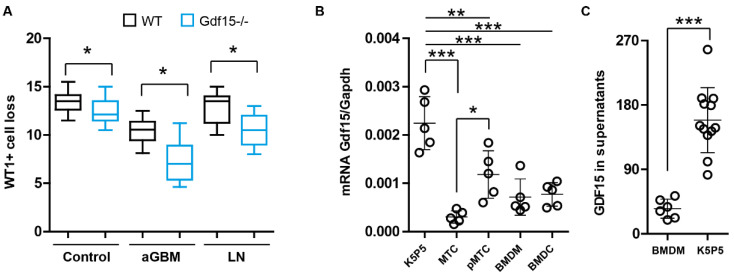
GDF15 expression in podocytes and their loss during kidney injury. (**A**): Kidneys of 6-month-old C57BL/6 and *Gdf15-/-* female mice (control), C57BL/6lpr and *Gdf15-/-lpr* (LN, lupus nephritis), and anti-glomerular basement membrane (anti-GBM) glomerulonephritis model were stained with antibodies against WT1 and pathological changes and the number of WT1+ cells (podocytes) quantified. Data are shown as box–whiskers plots or mean ± SD; * *p* < 0.05. (**B**): We isolated total RNA from various cell types such as podocytes (K5P5 cell line), mouse renal proximal tubular epithelial cells (MTCs, also known as MRPTEpiC), primary mouse renal tubular cells (pMTCs), bone-marrow-derived macrophages (BMDMs), and bone-marrow-derived dendritic cells (BMDCs) for qPCR analysis of *Gdf15* transcript. Data are presented as mean ± SD; * *p* < 0.05; ** *p* < 0.01; and *** *p* < 0.001. (**C**): GDF15 protein levels were determined in BMDMs and K5P5 podocytes supernatants using ELISA. Data are presented as mean ± SD; *** *p* < 0.001.

In *Gdf15* knockout mice, WT-1 positive cells were significantly reduced in glomeruli as compared to wild-type (WT) mice (Figure 1A). This was even more pronounced in kidneys from *Gdf15* knockout mice in glomerulonephritis models of anti-GBM and lupus nephritis (LN) (Figure 1A), suggesting that GDF15 may play a critical role in maintaining the integrity of the glomerular filtration barrier, podocyte survival, and glomerular inflammation. To determine which cells express and produce GDF15, we determined the mRNA expression levels of *Gdf15* in various cell types and measured the concentration of GDF15 in cell culture supernatants. We found that podocytes (K5P5 podocyte cell line) express high mRNA levels of *Gdf15* as compared to mouse renal proximal tubular epithelial cells (MRPTEpiC), primary mouse renal tubular cells (pMTC), bone-marrow-derived macrophages (BMDMs), and bone-marrow-derived dendritic cells (BMDCs) (Figure 1B). This finding was confirmed by an ELISA analysis showing that GDF15 is indeed produced specifically by podocytes (Figure 1C).

These findings collectively suggest that GDF15 plays a pivotal role in preserving the glomerular filtration barrier. The significant reduction of WT-1 positive cells in aged *Gdf15* knockout mice and severe models of glomerulonephritis underscores the importance of GDF15 in mitigating glomerular injury. Additionally, the high expression of *Gdf15* and specific production of GDF15 by podocytes suggest their contribution to renal homeostasis.

### 3.2. PAN Induces Gdf15 In Vitro and Gdf15 Knockout Alters Podocyte Expression Pattern

**Figure 2:**
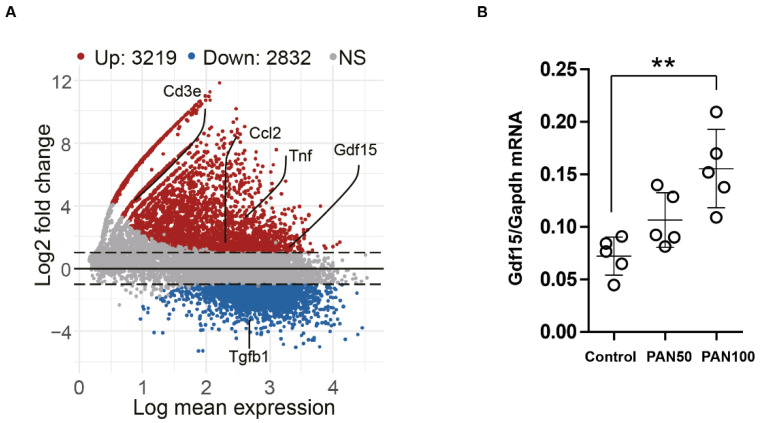
PAN induce Gdf15 expression in podocytes. (**A**): MA plots were created to display the shrink log2-fold change of genes for PAN-induced podocytes injury. Genes with differential expression and an adjusted *p*-value lower than 0.05 are identified by the colors red (high expression in PAN group) or blue. (**B**): Total mRNA of *Gfd15* from K5P5 podocytes stimulated with 50 or 100 µg/mL PAN for 24 h were analyzed with qPCR analysis. Data are presented as mean ± SD; ** *p* < 0.01.

To look at the expression pattern of *Gdf15* in podocytes during kidney injury, we made use of online available RNA sequencing data (GSE124622) and found that Gdf15 was induced by using PAN (100 μg/mL) treatment for 48 h (Figure 2A) [27,28]. Our in vitro experiments confirmed that the expression of *Gdf15* in podocytes was low under physiological conditions. However, podocytes stimulated with PAN revealed the upregulation of *Gdf15* expression (Figure 2B).

**Figure 3:**
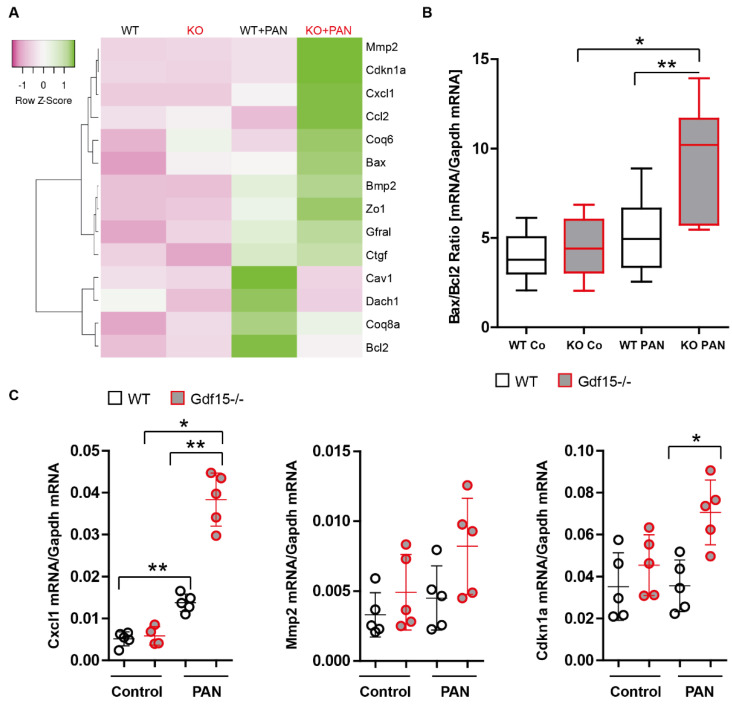
GDF15 controls the activation of podocytes upon PAN stimulation. We generated a knockout of GDF15 in podocytes using CRISPR/Cas9 technology. The specific gRNA was designed based on the DNA sequence of mouse *Gdf15* gene. The gRNA guide Cas9 cuts exon1 and disrupts the function of GDF15. Single cells were selected with cell sorting (GFP+). RT-PCR screening of single clones identified several KO clones. (**A**): GDF15 knockout or control (empty vector) cells were incubated for 18 h in the presence of 50 µg/mL of PAN. Total RNA was collected to quantify the gene expression levels by RT-PCR. Single clone RT-PCR results are presented in form of a heatmap. A heatmap shows altered genes from expression analysis of pre-selected transcripts. Genes indicated in green are upregulated and genes indicated in pink are downregulated to highlight differences between the samples. The rows are Z-Score scaled. The information about a single gene expression across the samples (controls and PAN) is given but the expression levels of gene X to gene Y cannot be concluded. Several genes that displayed significantly different expression between genotypes in the preliminary experiment were selected for real-time RT-qPCR validation. (**B**) *Bax/Bcl2* (n > 6 per group) and (**C**) *Cxcl1*, *Mmp2*, and *Cdkn1*. Data are presented as mean ± SD; * *p* < 0.05; ** *p* < 0.01.

We used the CRISPR/Cas9 technique to knock out *Gdf15* gene as a stable, long-term effect. We expected that the lack of GDF15 expression in podocytes mediated by CRIPSR/Cas9 would result in changes that contribute to the cellular susceptibility to PAN-induced injury than those observed with siRNA technology. To address this, we co-expressed Cas9 with the gRNA that specifically targets exon 1 of the mouse *Gdf15* gene in the K5P5 podocyte cell line. Multiple KO cell lines were selected and identified by screening for individual isolated clones using PCR followed by DNA sequencing. The negative ELISA results (no measurable values were detected) confirmed the absence of the GDF15 protein in these KO cell lines, which exhibited a normal growth and phenotype. Extended stimulation durations were employed in the experiments to not only capture alterations in the expression of chemokines, typically associated with rapid responses, but also to discern changes in cytoskeletal dynamics and cell death processes. Following stimulation with PAN for 18 h, *Gdf15* knockout podocytes exhibited altered *bax/bcl2* mRNA expression (Figure 3A). The validity of the bax/bcl2-expression datasets from the heat maps was further confirmed by the expression analysis that included a larger number of samples (n > 6). Gene expression analysis of apoptosis-related genes showed that mRNA levels of *Bax* increased in *Gdf15*-silenced and knockout podocytes, while the anti-apoptotic factor *Bcl-2* was reduced (Figure 3B). Additionally, we observed significant increase in *Cxcl1* mRNA expression levels in *Gdf15* knockout podocytes upon PAN stimulation as compared to the WT control group (Figure 3C). The data suggest that *Gdf15* deficiency might regulate cell-death relevant transcripts and chemokine production in PAN-stimulated podocytes.

### 3.3. Gdf15-Deficiency Induces Endoplasmic Reticulum Stress and Podocyte Death In Vitro

**Figure 4:**
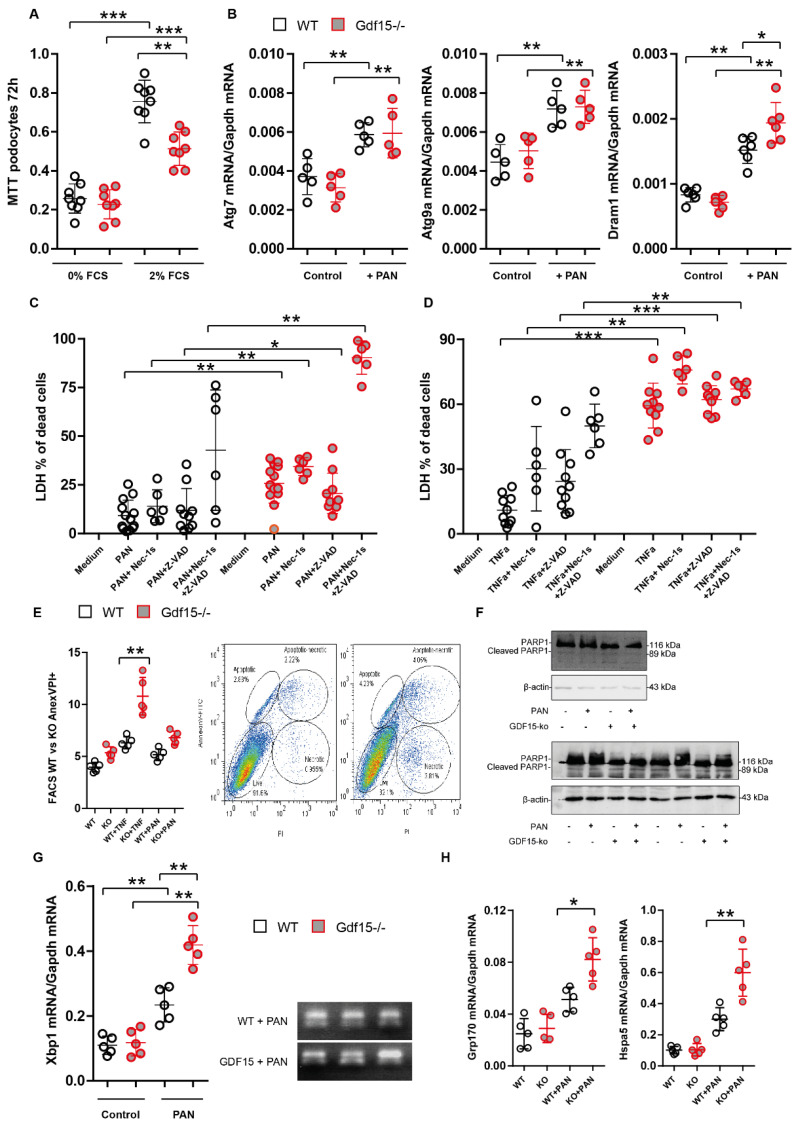
GDF15 protects from cell stress and cell death. (**A**): The metabolic activity of podocytes in the presence or absence of 2% FCS was evaluated by MTT assay after 72 h. The graphs represent a representative experiment (from three independent experiments) performed in a single run. (**B**): RNA was isolated from podocytes for RT-PCR analysis of autophagy-related genes. (**C**): Podocytes were untreated or exposed to PAN in the presence or absence of various cell death inhibitors for 24 h. Viability was evaluated by LDH assay. (**D**): Podocytes were untreated or exposed to TNFα and various cell death inhibitors for 24 h. Viability was evaluated by LDH assay. (**E**): Representative images of flow cytometry using Annexin V-FITC and PI double-stained cells. Cell apoptosis rates upon 50 µg/mL PAN and 500 ng/mL TNFα are presented as mean ± SD for at least three independent samples. The image of flow cytometry reading serves as a representation of the flow cytometry data and visualize the distribution of cells. (**F**): Protein expression levels of PARP1 protein forms were detected using Western blot analysis. (**G**): RNA was isolated from wild-type and knockout podocytes for RT-PCR analysis of *Xbp1* spliced version. Data are presented as mean ± SD; * *p* < 0.05; ** *p* < 0.01; and *** *p* < 0.001. (**H**): RNA was isolated from wild-type and knockout podocytes for RT-PCR analysis of *Xbp1*-dependent ER chaperones. Data are presented as mean ± SD; * *p* < 0.05; and ** *p* < 0.01.

To investigate whether GDF15 regulates the metabolism, autophagy, and cell death in injured podocytes, we cultured *Gdf15* knockout podocytes in the presence or absence of PAN and performed MTT and LDH assays, RT-PCR, immunoblotting and flow cytometry. As shown in Figure 4A, no difference in the metabolic activity between *Gdf15* knockout and control (WT) podocytes was observed under starving conditions (0% FCS), while the MTT values significantly decreased in *Gdf15* knockout podocytes compared with WT podocytes in the presence of 2% FCS, suggesting reduced cell viability due to *Gdf15* knockout in PAN-stimulated podocytes. Following up on the differential expression of the apoptosis-related genes *Bcl2/Bax* and the reduced metabolic activity, we further looked at autophagy and found that only *Dram1* mRNA expression levels but not *Atg7* or *Atg9a* appeared to be significantly affected by the lack of GDF15 in PAN-stimulated podocytes (Figure 4B). In addition, we performed LDH assays in the presence of the broad-spectrum caspase inhibitor Z-VAD and an inhibitor of necroptosis necrostatin-1 to identify through which form of cell death podocytes may die in response to PAN. Both PAN and TNFα induced significantly more cell death in *Gdf15* knockout podocytes compared to WT control podocytes (Figure 4C,D). Neither Z-VAD nor necrostatin-1 reduced the LDH release upon stimulation, indicating that caspase-dependent apoptosis and necroptosis are not the main forms of podocyte death. Moreover, Annexin V and PI staining indicated that the loss of GDF15 might initiate PAN-induced cell death. However, when we used strong inducers of cell death such as TNF-a, we observed a significant increase of apoptosis and necrosis in *Gdf15-/-* podocytes compared to wild-type cells (Figure 4E). In light of our findings, we further evaluated the overall cellular stress response, specifically focusing on mitochondrial stress and endoplasmic reticulum (ER) stress. We evaluated the cleavage of PARP1, which is a hallmark of parthanatos and has been shown to play a role in mitochondrial dysfunction [29]. However, we observed only minor differences in cleaved PARP-1, indicating that knockout cells display a slight resistance to PARP1-dependent cell death (Figure 4F). Furthermore, we examined the mRNA expression of *Xbp1* (X-box binding protein 1), a transcription factor that plays a critical role in the unfolded protein response (UPR) and coping with ER stress [30,31], and found that *Gdf15* knockout increased the expression of *Xbp1* in podocytes (Figure 4G). Since, XBP1 upregulates the expression of UPR target genes, including ER chaperones [32,33,34], we chose to evaluate the expression of *Grp170/Hyou1* and *Hspa5* that encodes ER-chaperone protein GRP78 and found the increased expression of these two factors in *Gdf15-/-* podocytes incubated with PAN (Figure 4H). Taken together, the data obtained from our experiments provides evidence suggesting that *Gdf15*-deficiency might lead to endoplasmic reticulum (ER) stress, and trigger podocyte death in vitro. The observed outcomes shed light on the crucial role of GDF15 in maintaining podocyte homeostasis.

### 3.4. GDF15-/- Mice Exhibit Mildly Enhanced Glomerular Immune Cell Infiltration after PAN Treatment

**Figure 5:**
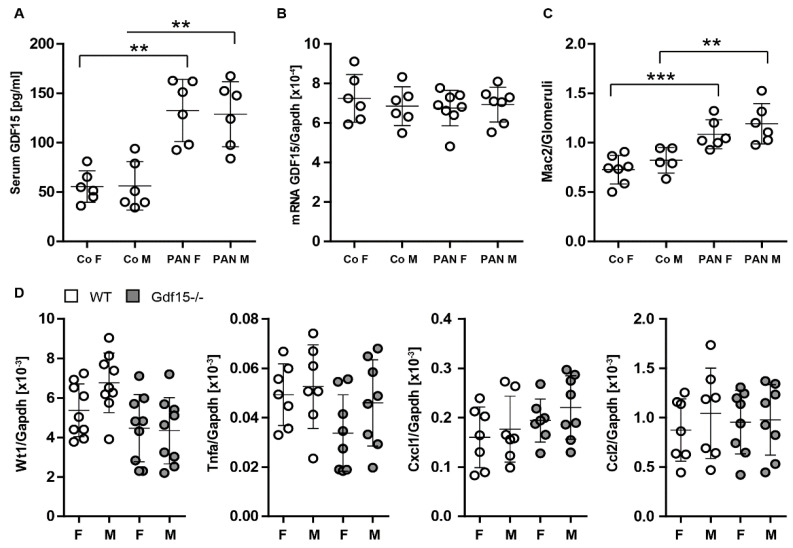
GDF15 expression in female (F) and male (M) mice during kidney injury. (**A**): We analyzed serum to quantify the levels of GDF15 protein. GDF15 serum protein (**A**) and Gdf15 kidney transcript (**B**) signatures did not differ in the male and female samples. (**C**): Kidneys of C57BL/6 male and female mice were stained with antibodies against Mac2; (**D**): RNA was isolated from C57BL/6 mice. Real-time PCR analysis represents relative expression of indicated genes. Data are presented as means ± SD; ** *p* < 0.01 *** *p* < 0.001.

Next, to assess the impact of gender on PAN-induced injury and systemic GDF15 levels, we compared male and female wild-type mice (Figure 5). Notably, our investigation did not reveal any significant differences in GDF15 production (Figure 5A), renal expression (Figure 5B), glomerular inflammation (Figure 5C), or renal gene expression (Figure 5D) between male and female mice aged 6–7 weeks following PAN treatment. Considering this lack of sex-dependent disparities in the parameters of interest, we decided to include both female and male mice. The obtained results demonstrate that the injection of PAN induces a glomerular influx of Mac2-positive cells as one of the hallmarks of inflammation. The lack of significance in gene expression may be attributed to the use of whole kidney tissue rather than dissected glomeruli. The mild effects of PAN are likely to be masked when analyzing the entire kidney, as the responses to PAN-induced injury within specific renal compartments may differ. Notably, the absence of observable podocyte loss suggests that the model primarily reflects glomerular inflammation with characteristics reminiscent of minimal change disease, indicating the subtle nature of podocyte injury. Furthermore, the elevated levels of GDF15 in the blood suggest a potential role for this factor in the observed pathology.

**Figure 6:**
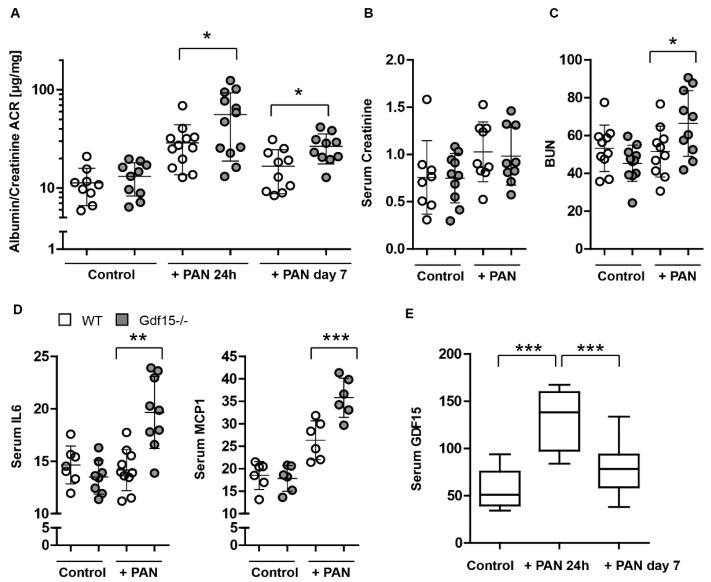
GDF15 protects mice from PAN-induced kidney injury. (**A**–**C**): Kidney function parameters including albumin/creatinine ratio ACR (**A**) were measured in the serum from 6-week-old WT and *Gdf15*-deficient mice 24 h and 7 days after PAN injection. Serum creatinine (**B**) and BUN (**C**) were measured in the serum from 6-week-old WT and *Gdf15*-deficient mice 7 days after PAN injection. (**D**): Serum IL-6 and MCP1 concentrations were measured on day 7 by ELISA. (**E**): GDF15 concentrations were measured after 24 h and on day 7 by ELISA. Data are presented as mean ± SD; * *p* < 0.05; ** *p* < 0.01; and *** *p* < 0.001.

In order to assess whether the observed effects in PAN-stimulated mouse podocytes could also be replicated in vivo, we injected *Gdf15* knockout and wild-type (WT) mice with 400 mg/kg PAN to induce podocyte injury and inflammation. Mice were sacrificed on day 7 and serum, urine, and kidney tissue collected after 24 h and on day 7 for analysis. As expected, PAN injection induced an increase in the albumin/creatinine ratio compared with the PBS control in WT mice (Figure 6A), although serum creatinine levels were unaffected in PAN-injected WT mice (Figure 6B). However, *Gdf15* knockout mice presented with a worsened kidney function as indicated by a significant increase in serum BUN levels (Figure 6C) and the albumin/creatinine ratio (Figure 6A) upon PAN injection as compared to WT mice, suggesting that the absence of GDF15 increases the risk for podocyte injury and kidney disease in mice. This was accompanied with an increase in the serum parameters of inflammatory markers such as IL-6 and MCP-1 in *Gdf15*-deficient mice compared with WT mice following PAN injection (Figure 6D). Similarly, serum GDF15 concentrations increased in WT mice following PAN injection (Figure 6E).

**Figure 7:**
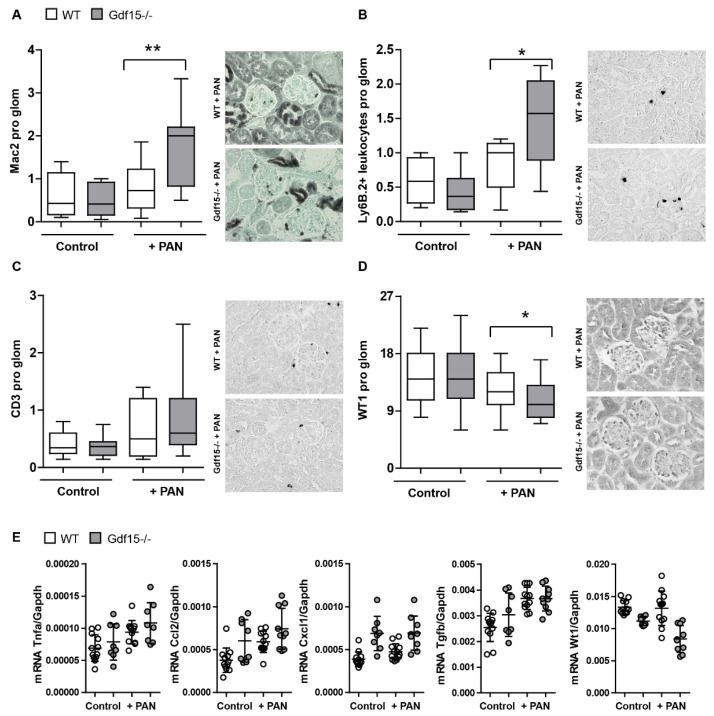
GDF15 reduces glomerular immune cell infiltration in PAN-induced kidney injury in mice. (**A**): Kidney sections from control and *Gdf15*-deficient mice were stained for (**A**) Mac-1+ glomerular macrophages, (**B**) Ly6B.2+ leukocytes, and (**C**) CD3+ T cells. The number of infiltrating cells was assessed in at least 20 glomeruli per kidney (n = 10 animals per group). (**D**): Kidneys were stained with the antibody against WT1. Podocytes were quantified by counting. The number of WT1+ cells (podocytes) was assessed in at least 20 glomeruli per kidney (n = 10 animals per group). Data are shown as box–whiskers plots ± SD. * *p* < 0.05, ** *p* < 0.01. (**E**): Relative mRNA expression of indicated genes from renal cortex were tested by RT-PCR.

To investigate the effect of GDF15 on the glomerular inflammatory response, we first examined the infiltration of immune cells in the glomeruli in PAN-induced kidney disease. Our histological analysis revealed an increased infiltration of Mac2+ macrophages (Figure 7A) and Ly-6B.2+ leukocytes (Figure 7B) in the glomeruli of Gdf15 knockout mice following PAN treatment as compared to WT mice, while the number of CD3+ T cells in the glomeruli was unaffected (Figure 7C). Quantification of glomerular WT1+ cells showed a significant reduction of podocytes in *Gdf15*-deficient mice compared with WT mice after PAN injection (Figure 7D). Additionally, we performed gene expression profiling of genes related to fibrosis and inflammation in the renal cortex using RT-qPCR. As shown in Figure 7E, only minor alterations in the mRNA expression levels of chemokines such as *Cxcl1* and *Ccl2*, and the cytokine *Tnfα* were observed in the cortex between *Gdf15*-deficient and WT mice following PAN-induced podocyte injury.

**Figure 8:**
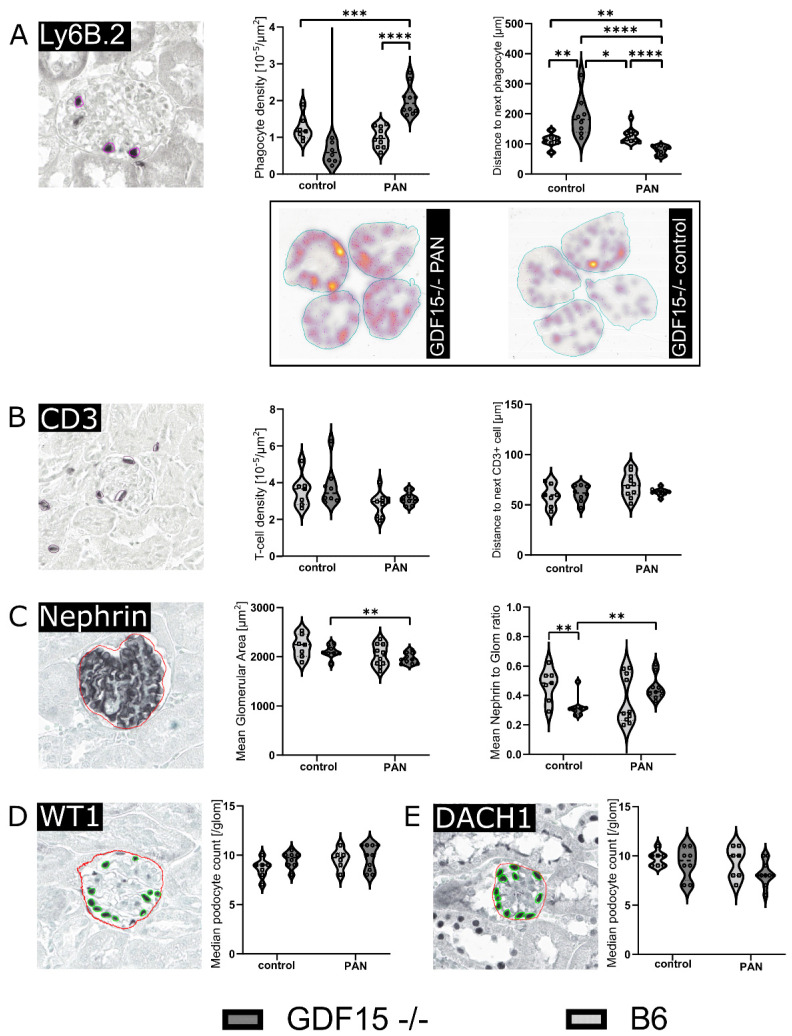
Deep-learning-assisted whole-slide kidney morphometry. Single-cell and glomerular analysis using Ly6B.2, CD3, Nephrin, and WT1 immunohistochemistry staining. (**A**): Leukocytes (Ly6B.2+ cells) invade the whole kidney in *Gdf15*-deficient mice after PAN-induced injury indicated by leukocyte density and distribution analysis. Representative whole-slide neutrophil heatmap images of *Gdf15-/-* mice with PAN treatment and no treatment (control) are shown. (**B**): Kidney sections from *Gdf15*-deficient (n = 7–10 as indicated by dots within the violin diagram) and WT (n = 7–10 as indicated by dots within the violin diagram) mice with and without (control) PAN injection were stained for CD3 and the CD3+ T-cell abundance and distribution quantified. (**C**–**E**): Mean glomerular area (framed) and mean nephrin to glom ratio (**C**), mean WT1+ podocyte (**D**), and DACH1+ podocyte numbers (**E**) in the glomerulus were analyzed. Morphometric results were obtained using a deep-learning-assisted segmentation approach in whole-slide images. Data are shown as violin-plots ± SD. * *p* < 0.05, ** *p* < 0.01, *** *p* < 0.001, and **** *p* < 0.0001. Each dot represents one representative kidney slide of one animal.

To analyze the cellular abundance and distribution in the glomeruli in more detail, we employed a deep-learning-assisted morphometry approach inspired by previously published works [35,36,37]. Consistent with our data above, we observed a strong infiltration of Ly6B.2+ leukocytes in the kidney of WT mice after PAN injection (Figure 8A) that was further aggravated in *Gdf15*-deficient mice (Figure 8A). No difference in the number of CD3+ T cells was found between the four groups (Figure 8B). When quantifying Nephrin+ glomeruli in kidney sections, we noticed a decrease in mean glomerular area in GDF15-deficient mice after PAN injection and an increase in the mean nephrin-to-glomerular-area ratio of *Gdf15*-deficient mice after PAN treatment (Figure 8C). Regarding WT1+ podocyte counts (Figure 8D) and DACH1+ podocyte counts (Figure 8E), we could not confirm the differences shown in Figure 7. Taken together, PAN promoted leukocyte infiltration in *Gdf15*-deficient mice and led to a decrease in mean glomerular area indicating glomerular damage. However, deep-learning morphometry on immunohistochemistry slides was not able to confirm in vivo podocyte injury in this mild model of glomerular disease. For further studies, detailed imaging methods like super-resolution or electron microscopy might be applied. Deep-learning algorithms are widely employed in all fields of research. Here, an image segmentation algorithm is used to detect immunohistochemistry-stained nuclei (Ly6B.2, CD3, WT1, and DACH1) or cytoplasm (Nephrin) instances in whole-slide kidney specimens. In the training phase, the algorithms learn to segment the desired structures from manually annotated images. Subsequently, the algorithms are run on the remaining images, and the obtained segmentation data are used for morphometrical analysis. Here, the technology allows a time-efficient quantitative morphometrical analysis that would be too time-consuming and expensive for humans. However, challenges persist, particularly concerning staining inhomogeneity, which poses a greater challenge for deep-learning algorithms compared to human observers.

## 4. Discussion

We had hypothesized that GDF15 has a protective effect on podocytes during glomerular injury. The mouse model of PAN-induced podocyte injury revealed that, indeed, GDF15 demonstrates a protective effect on kidney disease by mitigating the infiltration of immune cells into glomeruli and reducing proteinuria. In parallel, our in vitro experiments provide mechanistic insights into the protective role of GDF15. Specifically, GDF15 reduces inflammation, endoplasmic reticulum stress, and cell death in cultured podocytes. This suggests that GDF15 may exert its protective function at the molecular and cellular levels, contributing to the overall preservation of podocyte integrity.

Currently, the precise molecular mechanisms governing podocyte homeostasis and its impact on the glomerular environment are not fully understood. Given the close proximity of endothelial and mesangial cells with podocytes in the glomerulus, it is likely that podocytes significantly participate in homeostasis within the glomerulus and affect mesangial matrix expansion and glomerulosclerosis. The global genetic deletion of *Gdf15* did not result in any spontaneous renal insufficiency or significant proteinuria. However, we investigated the susceptibility of *Gdf15* knockout mice to podocyte injury induced by PAN, which causes morphological deformities and cytoskeletal disruptions leading to the effacement of podocytes both in vitro and in vivo [38,39]. The PAN-induced nephrotic syndrome in rats has been widely used to study the structural and functional alterations of the filtration barrier responsible for proteinuria, mimicking clinical manifestations in humans. Although mice generally exhibit resistance to PAN-induced nephrosis, transient proteinuria has been observed in certain mouse models with specific genetic backgrounds, such as podocyte-specific overexpression of cyclooxygenase-2 driven by the nephrin promoter [40], as well as in hypercholesterolemic mice lacking apolipoprotein E [41]. In our experimental settings, PAN was able to induce renal phenotypes; however, the associated glomerular inflammation and podocyte loss were only minimally increased. The limited pathological changes might be related to the fact that PAN initiates a mild model of kidney injury. Moreover, the loss of WT1+ podocytes could not be confirmed by the AI-based analysis. We believe that, in certain scenarios, manual pathology analysis offers several advantages over AI-based algorithms. Firstly, human pathologists can adapt their analysis to accommodate staining differences and subtle variations in sample preparation. Additionally, manual analysis allows for the incorporation of expert knowledge, adjustment of analysis, and contextual understanding of the tissue architecture, enabling the identification of abnormalities and artifacts that may indicate impurities or inconsistencies in the samples or unspecific bindings of antibodies. However, it is important to note that manual pathology analysis may introduce biases, which AI algorithms are designed to mitigate. It is of note that we exclusively relied on WT1 expression as the indicator of podocyte loss due to its specificity in regulating a wide array of target genes crucial for various physiological functions within podocytes. Nevertheless, we did not assess other significant proteins, such as nephrin, podocalyxin, podocin, P-cadherin, or WT-interacting proteins [42,43,44,45].

To reduce the influence of sex hormones on the results, we carefully considered the age of the mice, and evaluated the GDF15 levels and renal parameters in 6–7 weeks old animals that were used for the study. Previous studies reported an inverse correlation between the numbers of glomeruli and albuminuria, across different strains and sexes [46]. Conversely, estrogen may have a protective effect as certain potentially renoprotective genes contain predicted estrogen response elements, estrogen receptors are expressed on podocytes, and estradiol has been shown to attenuate podocyte apoptosis induced by puromycin or testosterone [47,48]. These differences did not apply in our experimental setting where only 6–7-week-old mice were used.

Podocyte injury is associated with mesangial cell proliferation and the infiltration of immune cells into glomeruli [49,50]. Therefore, understanding the role of GDF15 in modulating mesangial cell behavior and immune response could provide valuable insights into its potential therapeutic implications. Chen et al. demonstrated that the depletion of *Gdf15* resulted in the reduced proliferation of mesangial cells and activated autophagy in these cells. Their findings indicated the activation of the PI3K/AKT/mTOR signal upon *Gdf15* deficiency [51]. On the other hand, GDF15 is well known to regulate the magnitude of the inflammatory response of innate immune cells [18]. Further exploration of these pathways may pave the way for innovative interventions.

Our in vitro results indicate that GDF15 plays a role in stress signaling and regulates inflammation in podocytes, thereby protecting them from cell death. We have also observed certain aspects of significant endoplasmic reticulum (ER) stress in knockout podocytes following PAN treatment as indicated by *Xbp1* expression levels. During dysfunction of the ER, the IRE1α-XBP1 branch is highly dysregulated. IRE1α catalyzes the splicing of X-box-binding protein 1 (XBP1) mRNA, resulting in the production of the spliced form, XBP1s, which acts as a potent transcription factor initiating the unfolded protein response (UPR) program. We have observed increased levels of *Xbp1* in *Gdf15*-deficient podocytes upon PAN treatment. Consistent with our findings, Zhang et al. reported that *Gdf15*-deficient liver exhibits heightened ER stress, leading to increased XBP1s production, triglyceride accumulation, and the subsequent upregulation of *Fgf21* expression, potentially counteracting the effects of reduced PPARα levels [52]. Furthermore, the same study demonstrated that XBP1s activates *Gdf15* transcription in response to heightened ER stress by binding to its promoter [52]. The authors proposed that the disruption of GDF15 might impair fatty acid β-oxidation and ketogenesis, suggesting a disturbance in lipid metabolism homeostasis in podocytes as well. Mice with the podocyte-specific deletion of the genes encoding XBP1 and Sec63, a heat shock protein-40 chaperone required for ER protein folding, develop podocyte injury characterized by progressive albuminuria, foot process effacement, and compromised UPR, resulting in the decreased ability to mitigate damage by preventing the accumulation of misfolded proteins in the ER [53]. Furthermore, it has been demonstrated that the intact IRE1α-XBP1 pathway plays a cytoprotective role in glomerular diseases associated with podocyte injury [54,55]. In addition, in vitro treatment with PAN leads to a dose-dependent increase in ER stress markers in podocytes, triggering apoptosis and oxidative stress [56]. Our study revealed that chaperones implicated in responding to endoplasmic reticulum stress, such as Hspa5 or Grp170, were upregulated in *Gdf15*-deficient podocytes when stimulated with PAN. These chaperones have been previously identified to be upregulated during ER stress and are associated with XBP1 signaling [33,57,58]. The observed trends consistently indicate that GDF15 plays a crucial role in preventing cellular damage and maintaining cell viability. However, we did not observe many differences between wild-type and knockout cells in autophagy-related gene expression such as *Atg7* and *Atg9*. Autophagy, which is involved in the removal of misfolded proteins, appears to be a crucial pathway for maintaining podocyte health [59,60]. In support of our data, Pod-*Sec63/Xbp1* double-knockout mice did not exhibit different autophagosomal activity but still displayed severe ER stress [36]. This finding is surprising, considering that podocytes generally exhibit relatively high basal autophagy compared to other cell types [61,62]. Additionally, enhanced autophagy has been observed in glomerulopathies in humans, as indicated by elevated kidney ER stress in patients with MCD or FSGS [63,64]. Our study found that *Gdf15*-deficient podocytes exhibited significantly elevated levels of *Dram1*. However, it is important to note that this association may not be solely attributed to conventional autophagy. Although DRAM1 was initially recognized for its involvement in classical autophagy and apoptosis initiation [65,66], subsequent investigations have unveiled its role in alternative autophagy pathways as well [67]. Moreover, in numerous scenarios, the execution of apoptosis relies on autophagy. Particularly, in response to DNA damage, the expression of the macroautophagy regulator *Dram1* is crucial for p53-mediated apoptosis [65]. Thus, the heightened expression of *Dram1* in our study may signify ongoing or initiated apoptosis rather than alternative autophagy.

Our data demonstrate the increased susceptibility of *Gdf15*-deficient podocytes to cell death following PAN and TNF treatment. Using inhibitors such as Z-VAD and Necrostatin-1, we ruled out apoptosis and necroptosis as the primary mechanisms, yet we observed a significant elevation in the release of LDH, indicating cell damage. Additionally, we did not observe clear differences in the presence of cleaved PARP-1 protein, which is typically associated with parthanatos [68]. It is important to note that other forms of cell death could be at play when apoptosis and necroptosis are blocked [69,70]. These alternative forms of cell death may involve ER and oxidative stress. It is of note that the mechanisms underlying podocyte loss, including various sorts of cell death, as well as structural changes and detachment, are subject to ongoing debate. Nonetheless, the viability of *Gdf15*-deficient podocytes was compromised, warranting further investigation into the underlying mechanisms.

In conclusion, our findings provide support for the protective role of GDF15 in cellular stress following podocyte injury and contribute to a better understanding of how podocytes can mitigate stress, ultimately preserving the integrity of the glomerular filtration barrier. We used global *Gdf15*-deficient mice and cannot exclude the effects of other parenchymal and immune cells that are source of GDF15, discerning the specific effects solely attributed to podocytes [71,72,73,74]. Our study demonstrates the protective role of GDF15 in podocytes following injury, as evidenced by its ability to reduce inflammation and endoplasmic reticulum stress. GDF15 expression was higher in cultured podocytes than in other cell lines, and increased further upon PAN stimulation in vitro. Podocytes deprived of *Gdf15* showed more susceptibility to inflammatory and cell stress responses, as evidenced by the increased expression of cytokines, endoplasmic reticulum stress via elevated *Xbp1* expression, and increased cell death. Furthermore, PAN-induced renal injury in *Gdf15* knockout mice exhibited elevated glomerular immune cell infiltration alongside a phenotype of PAN-induced nephrosis as compared with wild-type mice. Additionally, PAN treatment resulted in only mild glomerular damage, yet GDF15 exhibited its anti-inflammatory properties, indicating its potential therapeutic value.

## Data Availability

All data resulting from our study are presented within the paper. Wemade use of online available RNA sequencing data in a Figure 2 (https://www.ncbi.nlm.nih.gov/geo/query/acc.cgi?acc=GSE124622) and included necessary information, such as dataset number (GSE124622) and references in the paper.
